# Longitudinal associations of loneliness with mental ill-health, physical ill-health, lifestyle factors and mortality in ageing adults in Thailand

**DOI:** 10.1186/s12888-023-05263-0

**Published:** 2023-11-17

**Authors:** Supa Pengpid, Karl Peltzer, Dararatt Anantanasuwong

**Affiliations:** 1 Department of Health Education and Behavioral Sciences, Faculty of Public Health, Mahidol University, Bangkok, Thailand; 2 Department of Public Health, Sefako Makgatho Health Sciences University, Pretoria, South Africa; 3 Department of Healthcare Administration, College of Medical and Health Science, Asia University, Taichung, Taiwan; 4 Department of Psychology, University of the Free State, Bloemfontein, South Africa; 5 Department of Psychology, College of Medical and Health Science, Asia University, Taichung, Taiwan; 6 Center for Aging Society Research (CASR), National Institute of Development Administration (NIDA), Bangkapi Bangkok, Thailand

**Keywords:** Loneliness, Mental health, Physical health, Health behaviour, Longitudinal study, Thailand

## Abstract

**Objectives:**

The aim of this study was to assess the longitudinal association between loneliness, mental and physical ill-health indicators, lifestyle factors and mortality among middle-aged and older adults in Thailand.

**Methods:**

We analyzed prospective cohort data of participants 45 years and older from three consecutive waves in 2015 (n = 5616), 2017 (n = 3600), and in 2020 (n = 2863) of the Health, Aging and Retirement in Thailand (HART) study. Loneliness was assessed with a single item. To assess the longitudinal associations between loneliness and health outcomes between 2015 (baseline), 2017 (first follow-up) and 2020 (second follow-up), we conducted Generalized Estimating Equations analysis (GEE).

**Results:**

The proportion of loneliness was 21.6% in 2015, 23.8% in 2017 and 21.3% in 2020. In the adjusted GEE logistic regression model, loneliness was positively associated with mental ill-health (poor self-rated mental health status, poor quality of life/happiness, depressive symptoms, and insomnia symptoms), physical ill-health (poor self-rated physical health status, hypertension, kidney disease, osteoporosis, and ADL disability), and lifestyle factors (physical inactivity, and having underweight). Furthermore, in adjusted Cox proportional hazards regression, loneliness was associated with mortality. In adjusted logistic regression, compared to without loneliness in all three study waves, having loneliness in one wave and/or two to three waves was positively associated with incident mental ill-health (incident poor self-rated mental health status, incident poor quality of life/happiness, incident depressive symptoms, and incident insomnia symptoms), incident physical ill-health (incident poor self-rated physical health status, incident diabetes, incident kidney disease, and incident ADL disability), and incident lifestyle factors (having incident underweight).

**Conclusion:**

We found that loneliness was associated with several mental and physical ill-health indicators, lifestyle factors and mortality. Enhanced screening and treatment of loneliness may reduce mental and physical ill-health indicators in Thailand.

**Supplementary Information:**

The online version contains supplementary material available at 10.1186/s12888-023-05263-0.

## Introduction

Loneliness is common in the general population and can increase in the ageing population due to a reduction of social relations [1–3]. The prevalence of loneliness among older adults in high-income countries was 28.5% [4], and in middle-income countries, for example, 9.9% in South Africa [5], 32.5% in Malaysia [6], 33.8% in India [7], and 21.7% in Thailand [8]. Loneliness can be considered as a social construct and not a mental disorder, such as depression, although reviews have shown that in older adults, loneliness was positively associated with depressive symptoms [9] and the onset of depression [10]. One other study showed that “loneliness is associated with depressive affect, but not with most other symptoms of depression” [11].

Loneliness may contribute to mental and physical ill-health [12, 13]. In terms of mental ill-health, loneliness has been associated with mental ill-health [14], depression [10, 15, 16], sleep disruption [13, 17–20], low life satisfaction [17, 21], and lower subjective well-being [13, 14]. Regarding physical ill-health, loneliness increased the odds of poor self-rated health [17, 22–24], cardiovascular disease [21, 25], hypertension, lung disease [25], diabetes [23], and functional disability [21, 22, 26]. Many investigations showed that loneliness was positively associated with engaging in an unhealthy lifestyle, such as physical inactivity [17, 21, 23, 27], current tobacco use [17, 18, 23, 24, 27], hazardous alcohol use [24], obesity [28], and underweight [21, 29]. Furthermore, in a systematic review loneliness showed an association with mortality [30].

Most research examining loneliness and its adverse health effects in older adults are conducted in high-income countries, but there is a lack of longitudinal information on these relationships in middle-income countries, such as Thailand [31, 32]. Some research seems to show differences between individualistic and collectivistic societies. For example, in a study among middle-aged and older adults in 14 European countries, Beller et al. [33] found that health effects of loneliness were stronger in more collectivistic countries and weaker in more individualistic countries. So, it is possible that in a more collectivistic Thailand health effects of loneliness are strong, which prompted this study.

Based on the cited research, we hypothesize that loneliness is associated with mental and physical ill-health, unhealthy behaviours, and mortality in middle-aged and older adults in Thailand. To improve our understanding on the association between loneliness and health outcomes in Thailand, the study aimed to assess the longitudinal associations between loneliness, and mental and physical ill-health indicators, lifestyle factors and mortality among ageing adults from 2015 to 2020 in Thailand.

## Methods

The longitudinal data of three consecutive waves of Thailand’s Health, Aging and Retirement (HART) study (2015, 2017 and 2020) were analyzed. In a national multi-step sampling design, one adult (45 years or older) was selected randomly per household; see details [34]. The trained field workers conducted face-to-face interviews in the home of the participants. In addition, during wave 2 and 3 exit interviews were conducted with proxy respondents (spouse or household members with the best information about the primary interviewee’s death, on the date, cause, and place of death). The “Ethics Committee in Human Research, National Institute of Development Administration – ECNIDA (ECNIDA 2020/00012)” approved the study protocol, and participants provided written informed consent. All experiments were performed in accordance with relevant guidelines and regulations (such as the Declaration of Helsinki).

### Measures

#### Exposure variable

*Loneliness* was measured from the Center for Epidemiologic Studies Depression Scale (CES-D-10) item, “In the past week, how often did you experience feeling lonely?” defined as “almost always (5–7 days), often (3–4 days) or sometimes (1–2 days)”=1 and “very rarely (less than one day) or none”=0 [35].

#### Outcome variables

#### Mental ill-health outcomes

*The self-rated mental health* s*tatus* was assessed with the question, “In general, how would you rate your mental health status?” reported on a 0 (= very poor) to 10 (= excellent) visual analogue scale. Self-rated poor mental health was defined as 0–7.0 (8.0 being the median).

*Quality of life or happiness* was sourced from the question, “In general, how satisfied are you with your quality of life (or how happy do you feel)?” reported on a 0 (= very poor) to 10 (= excellent) visual analogue scale. Self-rated poor quality of life/happiness was defined as 0–7 (8 being the median).

*Depressive symptoms* (≥ 10 scores) were evaluated using the CES-D-10 [35], without the loneliness item; Cronbach’s alpha was 0.7, in wave 1, 2, and 3 respectively.

*Insomnia symptoms* were defined as almost always (5–7 days) or often (3–4 days) (versus sometimes-1-2 days or very rarely or never) having trouble falling asleep/insomnia in the past week.

*Brain diseases, including dementia* were assessed by reported health care provider diagnosis.

#### Physical ill-health outcomes

*Self-rated physical health status* was measured with the item, “In general, how would you rate your physical health status?” reported on a 0 (= very poor) to 10 (= excellent) visual analogue scale. Self-rated (poor) physical health was defined as 0–6.0 (7.0 being the median).

Care-provider diagnosed chronic physical conditions including diabetes, kidney disease, hypertension, cardiovascular diseases, chronic lung disease, osteoporosis, and cancer.

*ADL disability* was sourced from a 4-item (dressing, washing, eating, and bathing) modified ADL scale [36]. Response options ranged from 0= “able to do it all by myself” to 3 = “need help for all steps”. ADL disability was defined as one of the four elements that cannot be done alone. (Cronbach’s α = 0.93 at wave 1, 0.90 at wave 2 and 0.92 at wave 3).

#### Life style factor outcomes

*Tobacco smoking* asked for, “Have you ever smoked cigarettes?” (“1 = yes, and still smoke now, 2 = yes, but quit smoking, and 3 = never”).

*Hazardous alcohol use* was sourced from questions on the amount and frequency of alcohol use, and defined as ≥ 3–4 and ≥ 5 standard units of alcoholic beverages for women and men, respectively, per week.

*Physical activity/exercise* (frequency: “How often do you exercise?” (days a week) and duration of any type: “On the day you exercise, how long do you exercise?” (minutes), was grouped into “none = inactivity, 1–149 min/week = low activity, and ≥ 150 min/week = high activity in the past week.” [37].

*Body Mass Index* (BMI) was sourced from self-reported body weight/height, and classified following Asian cut-offs criteria into “underweight (< 18.5 kg/m^2^), normal weight (18.5–22.9 kg/m^2^), overweight (23–24.9 kg/m^2^), obesity class I (25-29.9 kg/m^2^) and obesity class II (30 + kg/m^2^)” [38].

#### Mortality outcome

Mortality is measured by survival and exposure to death. The survival status was measured by the question of whether the respondent interviewed died in the 2015 wave or survived in the 2017 and 2020 waves.

#### Covariates

*Covariates* included sex, age, residence status, education, marital status, and subjective economic status (“How satisfied are you with your economic situation?” Rated from 1 to 10).

### Data analysis

Pearson Chi-square statistics were applied to compare sample characteristics across study years. Adjusted logistic regression was conducted between participants who dropped out and stayed in the study in relation to sociodemographic factors and health variables. To assess the longitudinal associations between loneliness and mental and physical ill-health and lifestyle factor outcomes between 2015 (baseline), 2017 (first follow-up) and 2020 (second follow-up), we conducted Generalized Estimating Equations analysis (GEE). GEE is a kind of regression analysis that examines the correlations between repeated measures in a person, including subjects regardless of missing values [39]. For the GEE analysis, the working correlation matrix structure was ‘Independent’, and the link function ‘logit’. Two models are presented for the development of each health outcome. The first model regressing loneliness on each health outcome is unadjusted, and in the second model adjustments are made for sociodemographic factors, mental and ill-health factors, and lifestyle factors for each health outcome. Covariates were selected based on previous research [10, 13–15, 25, 26, 30–32]]. Cox proportional hazards regression model was performed to assess the associations of loneliness with 5-year mortality in the total sample and calculate the hazard ratios (HRs) and 95% CI (model 1 unadjusted and model 2 adjusted with GEE model 2 covariates). Furthermore, logistic regression models were fitted between loneliness exposure and incident health outcomes (in wave 2 or 3, and free of condition in wave 1 or baseline). Collinearity was assessed with Variation Inflation Factors (VIFs) statistics but none was found. StataSE 15.0 (College Station, TX, USA) was used for the statistical analyses; *p* ≤ 0.02 was accepted as significant, missing values were discarded (< 3%), only body mass index had 6.0% missing, and was imputed.

## Results

Sample characteristics of the three study assessments in 2015, 2017 and 2020 are shown in Table 1. The proportion of participants 70 years and older increased from 40.6% to 2015 to 50.3% in 2020, and the proportion of male participants decreased from 47.8% to 2015 to 42.8% in 2015. The proportion of loneliness was 21.6% in 2015, 23.8% in 2017 and 21.3% in 2020. There were significant differences in lifestyle measures (hazardous alcohol use, tobacco smoking, and physical inactivity) and mental ill-health factors (probable depression, self-reported poor mental health, poor quality of life/happiness, brain disease/dementia and loneliness). Physical ill-health conditions (hypertension, diabetes, cardiovascular disease, chronic lung disease, osteoporosis, kidney disease, ADL disability) all significantly increased from 2015 to 2020. Of 5616 participants at baseline, 361 died, 336 refused and 2056 were not traced from 2015 to 2020 (see Table 1). Logistic regression shows the differences between participants who dropped out and stayed in the study in relation to sociodemographic factors and health variables. In terms of sociodemographic factors, participants who dropped out were older, were men, had higher education, lived in urban areas, were widowed and were Buddhists. From the 19 health indicators examined, only three (poor quality of life/happiness, having brain disease/dementia and those with physical inactivity) were higher in the drop out group than those who stayed in the study (see Supplementary Table 1).

**Table 1:** Descriptive statistics of the study variables over time, HART 2015–2020

Variables	Study year			P-value
	2015 (n = 5616)	2017 (n = 3600)	2020 (n = 2863)	
	N (%)	N (%)	N (%)	
**Sociodemographic factors**				
Age (70 plus)	2282 (40.6)	1628 (45.2)	1441 (50.3)	< 0.001
Sex (male)	2686 (47.8)	1653 (45.9)	1224 (42.8)	< 0.001
Education (> elementary)	1024 (18.3)	686 (19.2)	470 (16.6)	0.144
Residence (rural)	3008 (53.6)	1804 (50.1)	1375 (48.0)	< 0.001
Marital status (widowed)	1673 (30.4)	1032 (28.7)	937 (32.8)	0.002
Subjective economic status (low)	1588 (29.3)	1351 (39.4)	978 (35.7)	< 0.001
Religion (Buddhist)	5208 (92.9)	3273 (91.5)	2585 (91.1)	0.007
**Mental ill-health**				
Self-reported poor mental health	1647 (30.0)	1146 (31.0)	741 (23.5)	< 0.001
Poor quality of life/happiness	1689 (31.6)	1466 (41.3)	1074 (34.2)	< 0.001
Probable depression	581 (11.3)	296 (8.3)	127 (4.4)	< 0.001
Insomnia symptoms	911 (16.4)	545 (15.2)	336 (11.7)	< 0.001
Brain disease/dementia	47 (0.8)	53 (1.5)	37 (1.3)	0.013
**Loneliness**	1195 (21.6)	852 (23.8)	610 (21.3)	0.024
**Physical ill-health**				
Poor self-rated physical health status	1527 (27.8)	1255 (34.9)	734 (25.6)	< 0.001
Hypertension	1951 (34.7)	1463 (40.6)	1303 (45.5)	< 0.001
Cardiovascular disease	277 (4.9)	213 (5.9)	195 (6.8)	< 0.001
Kidney disease	105 (1.9)	123 (3.4)	123 (4.3)	< 0.001
Diabetes	849 (15.1)	571 (15.9)	543 (19.0)	< 0.001
Osteoporosis	187 (3.3)	132 (3.7)	175 (6.1)	< 0.001
Chronic lung disease	49 (0.9)	58 (1.6)	42 (1.5)	0.003
ADL disability	207 (3.8)	208 (5.6)	222 (7.0)	< 0.001
Cancer	29 (0.5)	35 (1.0)	44 (1.5)	< 0.001
**Lifestyle factors**				
Current tobacco smoking	706 (12.6)	490 (13.2)	341 (10.8)	< 0.001
Hazardous alcohol use	201 (3.6)	236 (6.4)	59 (1.9)	< 0.001
Physical inactivity	3288 (59.5)	1689 (45.8)	1590 (50.4)	< 0.001
Body mass index (BMI)-underweight^a^	563 (10.0)	363 (10.1)	327 (11.4)	0.108
BMI-obesity class II^a^	361 (6.4)	270 (7.5)	220 (7.0)	0.135

^a^Since 6.0% were missing on the original BMI, BMI was imputed

### Longitudinal analyses with health outcomes and loneliness as exposure variable

Table 2 shows the Odds Ratios of loneliness from separate regressions for each health outcome. In the GEE logistic regression model, adjusted for sociodemographic factors, lifestyle factors, mental ill-health factors, and physical ill-health factors, loneliness was positively associated with mental ill-health (poor self-rated mental health status, poor quality of life/happiness, depressive symptoms, and insomnia symptoms), physical ill-health (poor self-rated physical health status, hypertension, kidney disease, osteoporosis, and ADL disability), and lifestyle factors (physical inactivity, and having underweight). Furthermore, in adjusted Cox proportional hazards regression, loneliness was associated with mortality (see Table 2).

**Table 2:** Longitudinal associations between loneliness and health indicators

Outcome variables	Lone-liness	Model 1: unadjusted odds ratio (95% CI)	p-value	Model 2: adjusted odds ratio (95% CI)^a^	p-value
**Mental ill-health**					
Poor self-rated mental health status	No Yes	1 Reference 2.23 (2.03 to 2.44)	< 0.001	1 Reference 1.70 (1.52 to 1.90)	< 0.001
Study wave			Wave 1 Wave 2 Wave 3	1 (Reference) 0.89 (0.80 to 1.00) 0.64 (0.50 to 0.73	0.042 < 0.001
Poor quality of life/happiness	No Yes	1 Reference 2.20 (2.00 to 2.40)	< 0.001	1 Reference 1.57 (1.39 to 1.78)	< 0.001
Study wave			Wave 1 Wave 2 Wave 3	1 (Reference) 1.50 (1.36 to 1.67) 1.21 (1.07 to 1.36)	< 0.001 0.002
Depressive symptoms	No Yes	1 Reference 6.52 (5.79 to 7.35)	< 0.001	1 Reference 15.24 (12.90 to18.25)	< 0.001
Study wave			Wave 1 Wave 2 Wave 3	1 (Reference) 0.54 (0.45 to 0.65) 0.29 (0.23 to 0.37)	< 0.001 < 0.001
Insomnia symptoms	No Yes	1 Reference 2.40 (2.22 to 2.53)	< 0.001	1 Reference 2.30 (2.12 to 2.49)	< 0.001
Study wave			Wave 1 Wave 2 Wave 3	1 (Reference) 0.79 (0.70 to 0.90) 0.60 (0.52 to 0.70)	< 0.001 < 0.001
Brain disease/dementia	No Yes	1 Reference 1.71 (1.40 to 2.08)	< 0.001	1 Reference 1.28 (0.87 to 1.89)	0.213
Study wave			Wave 1 Wave 2 Wave 3	1 (Reference) 1.27 (0.83 to 1.94) 1.46 (0.92 to 2.32)	0.279 0.107
**Physical ill-health**					
Poor self-rated physical health status	No Yes	1 Reference 2.11 (1.93 to 2.31)	< 0.001	1 Reference 1.39 (1.23 to 1.57)	< 0.001
Study wave			Wave 1 Wave 2 Wave 3	1 (Reference) 1.28 (1.14 to 1.43) 0.83 (0.73 to 0.94)	< 0.001 0.005
Hypertension	No Yes	1 Reference 1.35 (1.23 to 1.48)	< 0.001	1 Reference 1.14 (1.02 to 1.27)	0.019
Study wave			Wave 1 Wave 2 Wave 3	1 (Reference) 1.21 (1.11 to 1.30) 1.40 (1.28 to 1.53)	< 0.001 < 0.001
Cardiovascular disease	No Yes	1 Reference 1.33 (1.18 to 1.51)	< 0.001	1 Reference 1.13 (0.93 to 1.37)	0.217
Study wave			Wave 1 Wave 2 Wave 3	1 (Reference) 1.12 (0.95 to 1.33) 1.38 (1.14 to 1.66)	0.176 < 0.001
Kidney disease	No Yes	1 Reference 1.46 (1.26 to 1.70)	< 0.001	1 Reference 1.51 (1.14 to 1.99)	0.005
Study wave			Wave 1 Wave 2 Wave 3	1 (Reference) 1.58 (1.23 to 2.03) 2.16 (1.65 to 2.83)	< 0.001 < 0.001
Diabetes	No Yes	1 Reference 1.17 (1.08 o 1.27)	< 0.001	1 Reference 1.09 (0.95 to 1.24)	0.212
Study wave			Wave 1 Wave 2 Wave 3	1 (Reference) 0.89 (0.81 to 0.98) 1.09 (0.97 to 1.21)	0.017 0.152
Osteoporosis	No Yes	1 Reference 1.72 (1.41 to 2.09)	< 0.001	1 Reference 1.34 (1.08 to 1.16)	0.009
Study wave			Wave 1 Wave 2 Wave 3	1 (Reference) 0.95 (0.75 to 1.20) 1.86 (1.49 to 2.32)	0.660 < 0.001
Chronic lung disease	No Yes	1 Reference 1.22 (0.94 to 1.58)	0.140		
ADL disability	No Yes	1 Reference 2.03 (1.84 to 2.23)	< 0.001	1 Reference 2.16 (1.78 to 2.62)	< 0.001
Study wave			Wave 1 Wave 2 Wave 3	1 (Reference) 1.60 (1.27 to 2.00) 2.41 (1.93 to 3.01)	< 0.001 < 0.001
Cancer	No Yes	1 Reference 1.17 (0.73 to 1.89)	0.512		
**Lifestyle factors**					
Current tobacco smoking	No Yes	1 Reference 0.88 (0.79 to 0.99)	0.032	1 Reference 0.95 (0.80 to 1.13)	0.547
Study wave			Wave 1 Wave 2 Wave 3	1 (Reference) 1.12 (0.99 to 1.26) 1.03 (0.89 to 1.18)	0.061 0.726
Hazardous alcohol use	No Yes	1 Reference 0.95 (0.80 to 1.13)	0.579		
Physical inactivity	No Yes	1 Reference 1.15 (1.08 to 1.23)	< 0.001	1 Reference 1.18 (1.06 o 1.31)	0.002
Study wave			Wave 1 Wave 2 Wave 3	1 (Reference) 0.55 (0.51 to 0.60) 0.69 (0.63 to 0.76)	< 0.001 < 0.001
Body mass index (BMI)-obesity class II	No Yes	1 Reference 1.15 (0.97 to 1.36)	0.117		
BMI-underweight	No Yes	1 Reference 1.42 (1.24 to 1.63)	< 0.001	1 Reference 1.27 (1.10 to 1.48)	< 0.001
Study wave			Wave 1 Wave 2 Wave 3	1 (Reference) 0.92 (0.81 to 1.05) 1.10 (0.96 to 1.27)	0.201 0.180
		Model 1: unadjusted Hazard Ratio (95% CI)		Model 2: adjusted Hazard Ratio (95% CI)	
**Mortality**		1 Reference 1.58 (1.27 to 1.98)	< 0.001	1 Reference 1.66 (1.24 to 2.22)	< 0.001

^a^Adjusted for age group, sex, education, marital status, subjective economic status, area of residence, religion, and all variables in the table; ***p < 0.001; **p < 0.01; *p < 0.05; ADL: Activities of Daily Living; CI: Confidence Interval;

### Longitudinal associations between loneliness and incident health indicators

In adjusted logistic regression, compared to without loneliness in all three study waves, having loneliness in one wave and/or two to three waves was positively associated with incident mental ill-health (incident poor self-rated mental health status, incident poor quality of life/happiness, incident depressive symptoms, and incident insomnia symptoms), incident physical ill-health (incident poor self-rated physical health status, incident diabetes, incident kidney disease, and incident ADL disability), and incident lifestyle factors (having incident underweight) (see Table 3).

**Table 3:** Longitudinal associations between loneliness and incident health indicators

Outcome variables	Loneliness	Model 1: unadjusted odds ratio (95% CI)	p-value	Model 2: adjusted odds ratio (95% CI)^a^	p-value
**Mental ill-health**					
Incident poor self-rated mental health status	0 wave 1 wave 2–3 waves	1 Reference 1.61 (1.32 to 1.97) 2.88 (2.16 to 3.85)	< 0.001 < 0.001	1 Reference 1.53 (1.24 to 1.88) 2.65 (1.96 to 3.58)	< 0.001 < 0.001
Incident poor quality of life/happiness	0 wave 1 wave 2–3 waves	1 Reference 1.67 (1.36 to 2.04) 2.38 (1.73 to 3.26)	< 0.001 < 0.001	1 Reference 1.62 (1.31 to 2.00) 2.33 (1.67 to 3.23)	< 0.001 < 0.001
Incident depressive symptoms	0 wave 1 wave 2–3 waves	1 Reference 8.36 (5.44 to12.85) 23.66 (14.92 to 37.53)	< 0.001 < 0.001	1 Reference 9.32 (5.92 to 14.69) 26.15 (15.98 to 42.80)	< 0.001 < 0.001
Incident insomnia symptoms	0 wave 1 wave 2–3 waves	1 Reference 1.85 (1.48 to 2.30) 3.11 (2.34 to 4.14)	< 0.001 < 0.001	1 Reference 1.87 (1.49 to 2.35) 2.98 (2.21 to 4.03)	< 0.001 < 0.001
Incident brain disease/dementia	0 wave 1 wave 2–3 waves	1 Reference 1.30 (0.70 to 2.39) 1.79 (0.87 to 3.71)	0.408 0.116		
**Physical ill-health**					
Incident poor self-rated physical health status	0 wave 1 wave 2–3 waves	1 Reference 1.58 (1.29 to 1.92) 2.41 (1.81 to 3.20)	< 0.001 < 0.001	1 Reference 1.44 (1.17 to 1.77) 2.12 (1.57 to 2.86)	< 0.001 < 0.001
Incident hypertension	0 wave 1 wave 2–3 waves	1 Reference 1.15 (0.92 to 1.44) 1.17 (0.84 to 1.62)	0.213 0.350		
Incident cardiovascular disease	0 wave 1 wave 2–3 waves	1 Reference 1.28 (0.91 to 1.79) 1.57 (1.03 to 2.41)	0.162 0.038	1 Reference 1.27 (0.89 to 1.81) 1.55 (0.98 to 2.44)	0.181 0.089
Incident kidney disease	0 wave 1 wave 2–3 waves	1 Reference 1.72 (1.17 to 2.54) 2.10 (1.31 to 3.37)	0.006 0.002	1 Reference 1.59 (1.07 to 2.38) 1.82 (1.11 to 3.01)	0.023 0.018
Incident diabetes	0 wave 1 wave 2–3 waves	1 Reference 1.33 (0.98 to 1.80) 1.66 (1.13 to 2.44)	0.068 0.009	1 Reference 1.34 (0.98 to 1.82) 1.64 (1.10 to 2.45)	0.072 0.015
Incident osteoporosis	0 wave 1 wave 2–3 waves	1 Reference 1.41 (1.04 to 1.92) 1.28 (0.86 to 1.95)	0.026 0.243	1 Reference 1.27 (0.93 to 1.74) 1.05 (0.68 to 1.62)	0.139 0.831
Incident chronic lung disease	0 wave 1 wave 2–3 waves	1 Reference 0.98 (0.53 to 1.80) 1.09 (0.49 to 2.42)	0.942 0.835		
Incident ADL disability	0 wave 1 wave 2–3 waves	1 Reference 1.85 (1.37 to 2.49) 3.01 (2.12 to 4.25)	< 0.001 < 0.001	1 Reference 1.22 (1.04 to 1.98) 2.32 (1.59 to 3.37)	0.026 < 0.001
Incident cancer	0 wave 1 wave 2–3 waves	1 Reference 1.62 (0.85 to 3.11) 1.41 (0.58 to 3.39)	0.145 0.447		
**Lifestyle factors**					
Incident current tobacco smoking	0 wave 1 wave 2–3 waves	1 Reference 0.94 (0.68 to 1.31) 0.80 (0.50 to 1.29)	0.725 0.361		
Incident hazardous alcohol use	0 wave 1 wave 2–3 waves	1 Reference 0.82 (0.58 to 1.14) 0.61 (0.37 to 1.02)	0.228 0.059		
Incident physical inactivity	0 wave 1 wave 2–3 waves	1 Reference 0.89 (0.69 to 1.15) 1.18 (0.83 to 1.18)	0.373 0.371		
Incident body mass index (BMI)-obesity class II	0 wave 1 wave 2–3 waves	1 Reference 1.35 (0.91 to 2.01) 1.70 (1.04 to 2.76)	0.141 0.034	1 Reference 1.41 (0.94 to 2.13) 1.66 (0.99 to 2.79)	0.101 0.056
Incident BMI-underweight	0 wave 1 wave 2–3 waves	1 Reference 1.20 (0.90 to 1.59) 2.19 (1.57 to 3.05)	0.224 < 0.001	1 Reference 1.08 (0.80 to 1.48) 1.87 (1.31 to 2.66)	0.628 < 0.001

^a^Adjusted for age group, sex, education, marital status, subjective economic status, area of residence, and religion; ***p < 0.001; **p < 0.01; *p < 0.05; ADL: Activities of Daily Living; CI: Confidence Interval

## Discussion

This study is the first to investigate the associations between loneliness and the longitudinal development of health outcomes in Thailand. We found that loneliness was positively associated with the prevalence and incidence of mental ill-health (poor self-rated mental health status, poor quality of life/happiness, depressive symptoms, and insomnia symptoms), the prevalence and/or incidence of physical ill-health (poor self-rated physical health status, hypertension, kidney disease, diabetes, osteoporosis, and ADL disability) and the prevalence and/or incidence of lifestyle factors (physical inactivity, and having underweight), and mortality.

Consistent with previous research [10, 13–21], we found that loneliness was positively associated with mental ill-health (poor self-rated mental health status, poor quality of life/happiness, depressive symptoms, and insomnia symptoms). Moreover, we found a positive association between loneliness and incident brain disease/dementia in univariable analysis, which is also in agreement with previous investigations [40, 41]. The study found high associations between loneliness and mental ill-health, which may be explained by its comorbidity with mental ill-health, such as depression, and may possibly be bidirectional with depressive symptoms [23]. Furthermore, loneliness can cause thoughts that cause anxiety and reduce the ability to relax, leading to symptoms of insomnia [42].

In line with previous research [17, 21–26] we found that loneliness was positively associated with the prevalence and/or incidence of physical ill-health (poor self-rated physical health status, hypertension, kidney disease, diabetes, osteoporosis, and ADL disability). The finding that loneliness was associated with various poor mental and physical ill-health outcomes in this population of middle-aged and older adults in Thailand, may confirm that health effects of loneliness are stronger in more collectivistic countries, such as Thailand, and weaker in more individualistic countries [33]. Mechanisms explaining the impact of loneliness on physical ill-health include neurobiological processes that generate cardiovascular and inflammatory stress responses [18] and persons with loneliness may engage in fewer health-promoting behaviours (e.g., physical inactivity, malnutrition) leading to higher physical ill-health [13].

Regarding lifestyle factors, we found in consistence with former studies [17, 21, 23, 27, 29], positive associations between loneliness and physical inactivity and underweight. The impact of loneliness on physical inactivity can be explained by the process of loneliness reducing affective self-regulation and contributing to reducing the motivation of participants to exercise [42]. However, while some previous research [17, 18, 23, 24, 27, 28] found associations between loneliness and current tobacco use, hazardous alcohol use and obesity, we did not find any significant associations. In unadjusted analysis, loneliness was negatively associated with current smoking. Aging adults in Thailand may not engage in substance use as a way to cope with loneliness [24, 43].

Study limitations.

The limitations of the study include that variables were evaluated by self-reporting, and that loneliness was only assessed with a single item, however high correlations with multiple item loneliness measures have been established [44], and the CES-D loneliness item “performs similarly to other loneliness measures” [45]. A further limitation includes the high loss at follow-up. However, from the 19 health indicators examined, only three (poor quality of life/happiness, having brain disease/dementia and those with physical inactivity) were higher in the drop out group than those who stayed in the study. Moreover, there is the potential of reverse causality and variables not assessed in this study may have influenced health outcomes. The study design used a national random sample of middle-aged and older adults in Thailand, however, age-standardized weighting to the national population was not conducted.

## Conclusion

We found that loneliness and/or degree of loneliness exposure was positively associated with the prevalence and incidence of mental ill-health (poor self-rated mental health status, poor quality of life/happiness, depressive symptoms, and insomnia symptoms), the prevalence and/or incidence of physical ill-health (poor self-rated physical health status, hypertension, kidney disease, diabetes, osteoporosis, and ADL disability) and the prevalence and/or incidence of lifestyle factors (physical inactivity, and having underweight), and mortality. This may confirm that in a collectivistic society in Thailand loneliness was associated with various adverse health outcomes. Enhanced screening and treatment of loneliness may reduce various negative health outcomes in Thailand.

### Electronic supplementary material

Below is the link to the electronic supplementary material.

Supplementary Material 1

## Data Availability

Data is publicly available at Health, Aging, and Retirement in Thailand (HART): https://hart.nida.ac.th/download-center/.
