# Supplementary material for: Longitudinal associations of loneliness with mental ill-health, physical ill-health, lifestyle factors and mortality in ageing adults in Thailand
Source: BMC Psychiatry. 2023 Nov 17;23:855. doi: 10.1186/s12888-023-05263-0 (PMC10656829; doi:10.1186/s12888-023-05263-0)
Supplement: Supplementary file 1 — Supplementary Material 1 [file 12888_2023_5263_MOESM1_ESM.docx]

Supplementary Table 1: Adjusted logistic regression results predicting study dropout by sociodemographic and health variables

| Variables | Adjusted odds ratio (95% CI)^a^ | P-value |
| --- | --- | --- |
|  |  |  |
| **Sociodemographic factors** |  |  |
| Age (70 plus) | 1.34 (1.17 to 1.54) | <0.001 |
| Sex (male) | 1.38 (1.21 to 1.58) | <0.001 |
| Education (>elementary) | 1.55 (1.32 to 1.82) | <0.001 |
| Residence (rural) | 1.39 (1.23 to 1.57) | <0.001 |
| Marital status (widowed) | 1.22 (1.06 to 1.42) | 0.007 |
| Subjective economic status (low) | 1.12 (0.98 to 1.29) | 0.111 |
| Religion (Buddhist) | 1.42 (1.12 to 1.80) | 0.004 |
| **Mental ill-health** |  |  |
| Self-reported poor mental health | 1.03 (0.88 to 1.21) | 0.687 |
| Poor quality of life/happiness | 1.28 (1.11 to 1.48) | <0.001 |
| Probable depression | 1.20 (0.96 to 1.51) | 0.114 |
| Insomnia symptoms | 1.02 (0.86 to 1.22) | 0.794 |
| Brain disease/dementia | 2.34 (1.11 to 4.95) | 0.026 |
| **Loneliness** | 0.91 (0.77 to 1.07) | 0.252 |
| **Physical ill-health** |  |  |
| Poor self-rated physical health status | 0.92 (0.77 to 1.06) | 0.196 |
| Hypertension | 0.96 (0.84 to 1.10) | 0.546 |
| Cardiovascular disease | 0.88 (0.66 to 1.18) | 0.393 |
| Kidney disease | 1.22 (0.78 to 1.90) | 0.385 |
| Diabetes | 1.05 (0.88 to 1.25) | 0.581 |
| Osteoporosis | 0.74 (0.52 to 1.04) | 0.080 |
| Chronic lung disease | 0.93 (0.49 to 1.75) | 0.815 |
| ADL disability | 1.39 (0.98 to 1.33) | 0.068 |
| Cancer | 1.81 (0.78 to 4.19) | 0.165 |
| **Lifestyle factors** |  |  |
| Current tobacco smoking | 1.09 (0.99 to 1.33) | 0.370 |
| Hazardous alcohol use | 1.04 (0.76 to 1.44) | 0.547 |
| Physical inactivity | 1.28 (1.13 to 1.45) | <0.001 |
| Body mass index (BMI)-underweight^a^ | 1.16 (0.94 to 1.42) | 0.163 |
| BMI-obesity class II | 1.08 (0.84 to 1.38) | 0.561 |

^a^Adjusted for all variables in the table
